# Exploring the influence of context in a community-based facilitation intervention focusing on neonatal health and survival in Vietnam: a qualitative study

**DOI:** 10.1186/s12889-015-2142-2

**Published:** 2015-08-22

**Authors:** Duc M. Duong, Anna Bergström, Lars Wallin, Ha TT Bui, Leif Eriksson, Ann Catrine Eldh

**Affiliations:** Hanoi School of Public Health, 138 Giang Vo Street, Ba Dinh District, Ha Noi, Vietnam; International Maternal and Child Health (IMCH), Department of Women’s and Children’s Health, Uppsala University, SE-751 85 Uppsala, Sweden; Division of Global Health/IHCAR, Department of Public Health Sciences, Karolinska Institutet, Nobels vag 9, SE-171 77 Stockholm, Sweden; School of Education, Health and Social Studies, Dalarna University, SE-791 88 Falun, Sweden; Department of Neurobiology, Care Sciences and Society, Division of Nursing, Karolinska Institutet, SE-171 77 Stockholm, Sweden

## Abstract

**Background:**

In the Neonatal health – Knowledge into Practice (NeoKIP) trial in Vietnam, local stakeholder groups, supported by trained laywomen acting as facilitators, promoted knowledge translation (KT) resulting in decreased neonatal mortality. In general, as well as in the community-based NeoKIP trial, there is a need to further understand how context influences KT interventions in low- and middle-income countries (LMICs). Thus, the objective of this study was to explore the influence of context on the facilitation process in the NeoKIP intervention.

**Methods:**

A secondary content analysis was performed on 16 Focus Group Discussions with facilitators and participants of the stakeholder groups, applying an inductive approach to the content on context through naïve understanding and structured analysis.

**Results:**

The three main-categories of context found to influence the facilitation process in the NeoKIP intervention were: (1) Support and collaboration of local authorities and other communal stakeholders; (2) Incentives to, and motivation of, participants; and (3) Low health care coverage and utilization. In particular, the role of local authorities in a KT intervention was recognized as important. Also, while project participants expected financial incentives, non-financial benefits such as individual learning were considered to balance the lack of reimbursement in the NeoKIP intervention. Further, project participants recognized the need to acknowledge the needs of disadvantaged groups.

**Conclusions:**

This study provides insight for further understanding of the influence of contextual aspects to improve effects of a KT intervention in Vietnam. We suggest that future KT interventions should apply strategies to improve local authorities’ engagement, to identify and communicate non-financial incentives, and to make disadvantaged groups a priority. Further studies to evaluate the contextual aspects in KT interventions in LMICs are also needed.

## Background

In spite of the rapid increase of research findings, a corresponding translation of available relevant knowledge into practice does not occur [1]. Over the past two decades, numerous efforts to bridge the know-do gap, also referred to as efforts to increase knowledge translation (KT), have been actively initiated on national and international research agendas [2]. The field of KT aims to improve health services and strengthen the healthcare systems through “*a dynamic and iterative process that includes synthesis, dissemination, exchange and ethically sound application of knowledge*” ([3], para. 1). While there are implementation strategies known to be useful for KT in a certain context, no strategies have been found to be effective across all contexts [4]. Context, here defined as *“the environment or setting in which the proposed change is to be implemented” (*[5], p. 150), needs to be understood when implementing a particular KT strategy in different settings, or evaluating different outcomes of that strategy [6]. Further, it is suggested that a KT intervention is more likely to succeed if tailored to a specific context [7, 8], including for example sectoral, disciplinary, geographic, cognitive, and cultural factors [9, 10]. Hence, in order to better plan and evaluate the effect of KT interventions, a better understanding of which, and how, contextual factors influence the use of new and relevant knowledge is needed [11, 12]. Currently, for low- and middle-income countries (LMICs), this understanding is pending.

Globally, 99 % of the neonatal deaths (that is, deaths in the first 28 days of life) occur in LMICs and currently these deaths account for 44 % of the deaths of those aged under five years [13]. Up to two-thirds of the annual 2.9 million neonatal deaths [13] could be avoided with increased use of evidence-based and cost-effective practices [14]. However, these practices are too seldom successfully applied. Thus, there is a need to study various implementation strategies that will help to bridge the know-do gap [15]. Community-based interventions have been found to be effective in reducing neonatal mortality in several LMICs [16]; in particular, those interventions using facilitators to support women’s groups to identify and act on local problems [17, 18]. However, the evaluations of these interventions demonstrate that effect size varies largely. Although contextual aspects could help explain this variation, information about the context in which interventions are implemented is rarely reported, either from community-based interventions in general, or from facilitation interventions in particular [11].

For three years (2008–2011), a cluster-randomised controlled trial, NeoKIP (Neonatal health – Knowledge Into Practice), was conducted in the Quang Ninh province, located in northern Vietnam. Quang Ninh is classified as a low- and middle-income province, with an annual average income per capita recently reaching US$2,000 [19]. In 2008, Quang Ninh had more than one million inhabitants, with 8 % of the population belonging to what is defined as ‘poor’ households (households with an average income of less than US $240–$250 per capita per year) [19]. The terrain of the province varies, with 80 % covered by mountains and hills. The residents represent more than 10 ethnic groups, with the majority belonging to the Kinh group (accounting for about 80 % of the population) [19]. Most of the ethnic minority groups have their own language and culture, which differs from each other.

The NeoKIP trial evaluated the use of facilitators supporting local stakeholder groups for improved neonatal health and survival [20]. Eight districts with 90 communes were included in the trial where 44 communes were randomised to intervention and 46 to control. The trial recruited and trained local laywomen (*n* = 11) being members of the nation-wide organization, Women’s Union, who acted as facilitators in the intervention communes. In each of the intervention communes, a Maternal and Newborn Health Group (MNHG) was constituted. Each MNHGs consisted of 7–8 members: three commune health centre (CHC) staff; one population collaborator; one village health worker; one or two members of the Women’s Union (village and commune level); and a chairperson or a vice chairperson of the commune (who was responsible for health issues). Supported by their facilitator, each MNHGs held monthly meetings applying the quality improvement method, Plan-Do-Study-Act [21] to help MNHGs in identifying local problems within maternal and neonatal health and acting on those problems [20]. We hypothesized that the combination of community-based groups and support from facilitators would generate beneficial process of change influencing neonatal health and survival over time. In this process context factors could have a supportive and/or hindering impact.

By the third year of the NeoKIP trial, the risk of neonatal mortality was 49 % lower in the intervention communes than in the control communes [22]. Thus, this community-based intervention was proposed to be an effective strategy for improving neonatal health and survival in a low- and middle-income setting like Quang Ninh. According to the Promoting Action on Research Implementation in Health Services framework, successful implementation of KT interventions is a function of the interaction of context, facilitation, and evidence [8]. Previously, we have explored ‘facilitation’ as the implementation strategy in this setting [23]. Yet, with the limited knowledge of how context influences facilitation [24], there is a need to further explore what contextual aspects were present and how these aspects influenced the change process in communes involved in the NeoKIP project. Not only would an understanding of context indicate pros and cons of this particular facilitation intervention, but also of KT in similar settings. Thus, the aim of this study was to explore the influence of context on the facilitation process as described by the participants of the NeoKIP trial.

## Methods

### Study design

In order to enhance the understanding of ‘context’, a secondary analysis [25] was conducted of the Focus Group Discussions (FGDs) performed with all facilitators and a sample of MNHG members of the NeoKIP trial.

### Study setting and sample

As part of the NeoKIP trial, the FGDs were performed in the 44 intervention communes in Quang Ninh province, Vietnam. During the 3 years of intervention, 16 FGDs were conducted. These included four FGDs with all facilitators conducted over the intervention period (at 0, 6, 27, and 36 months). In addition, 6 MNHGs participated in 2 rounds of FGDs (21 and 36 months into the intervention), adding 12 FGDs with MNHGs. In each FGD, there were 7–8 participants. The 6 MNHGs partaking in the FGDs were purposely sampled representing a variation of groups regarding geographical locations, facilitators acting in the groups and group performance. Facilitators and the NeoKIP researchers jointly ascertained the group performance.

### Data collection

Semi-structured interview guides, with open-ended questions and probes [26], steered all FGDs, including experiences of the intervention and aspects hindering and/or facilitating KT. Two FGDs with facilitators were moderated by a Swedish researcher speaking in English, requiring simultaneous translation between English and Vietnamese and vice versa, while a native moderator performed the remaining 14 FGDs in Vietnamese. Each FGD lasted 60–120 min. All FGDs were audio recorded and transcribed verbatim. Due to a technical error, one FGD was not recorded, but the notes of the moderator were considered comprehensive enough to be included in the analysis. All Vietnamese texts were translated to English before analysis.

### Data analysis

The texts from the FGDs were analysed by inductive content analysis [27]. Initially, the corresponding author read and re-read the texts to become acquainted with the whole data set, providing for a naïve understanding of the idea of ‘context’. The naïve understanding of each interview was written down, and brought together in a naïve understanding of the whole data set. Subsequently, a structured analysis was performed, identifying all meaning units on contextual aspects, labelling them with unique codes. Subcategories were formed, and later, these were merged into categories. To conclude, the categories were formed as main-categories [27]. Throughout the analysis, the naïve understandings were used as a background to assure trustworthiness of the analysis [28]. The analysis process was completed by the first author and by two co-authors (ACE and AB) separately, and discussed until full agreement was reached.

## Ethical considerations

The NeoKIP trial (ISRCTN44599712) was approved by the Ministry of Health in Vietnam (ref 3934/QDBYT), and the Research Ethics Committee at the Uppsala University in Sweden (ref 2005:319).

## Results

Overall, three main-categories on context were identified to influence the implementation of the NeoKIP intervention:

- Support and collaboration of local authorities and other communal stakeholders
- Incentives to, and motivation of, participants
- Low health care coverage and utilization

The three main-categories originated from the experiences of participants formed as 31 sub-categories and later 8 categories. An overview of the subcategories and the categories is provided in Table 1.

**Table 1:** Sub-categories, categories and main-categories on experiences of context in the NeoKIP intervention

Sub-categories	Categories	Main-categories
Knowing how to ensure support from authorities	Local authorities need to be involved	Support and collaboration of local authorities and other communal stakeholders
Support of local authorities ensures the running of the project		
Involvement of local authorities is crucial		
Decisions made in top-down processes		
Authorities help dealing with reimbursement issues		
Being supported by authorities assures the collaboration of communal stakeholders		
Collaboration with representatives of local organisations ensures the running of the project	Collaboration of other communal stakeholders is needed	
Collaboration among stakeholders supports the running of the project		
Increased health awareness influences users’ requests for health services	Users’ utilization of health services motivates health providers	Incentives to, and motivation of, participants
Appreciation and trust of users motivates health care providers		
Gaining new knowledge is a benefit of the running of the project	Recognition of benefits among MNHG members is needed	
Recognizing the project’s results helps running the project		
Strengthening relationship among individuals supports the running of the project		
Working in a multi-stakeholder group supports the running of the project		
Information sharing supports the running of the project		
Participants of “projects” expecting to be reimbursed	Reimbursement is important but can be balanced by a perceived importance	
Participants of “meetings” expecting to be reimbursed		
Money needed to run project in order to provide for transports and meals		
Recognizing project’s importance outweighs necessity to reimburse		
Enthusiasm of MNHG members supports the running of the project		
Sense of responsibility of MNHG members supports the running of the project		
Understanding cultural differences of the various ethnic groups is needed for running the project	Acknowledging the disadvantaged groups in society is important to reach the whole population	Low health care coverage and utilization
Language diversity is a barrier for communication		
Difficult weather hinders accessibility to health care facilities	Accessibility and affordability among users influence health care utilization	
Difficult transportation hinders accessibility to health care facilities		
Service fee influences people’s health care utilization		
Lack of professional competencies influences service provision	Lack of resources hinders health service provision	
Lack of time constrains the running of the project		
Infrastructure of health facilities influences service provision		
Lack of workforce hinders implementation of group activities		
In remote areas, lack of resources further hampers the implementation of health services		

### Support and collaboration of local authorities and other communal stakeholders

The participants recognized the importance of supporting, involving, and assuring a good collaboration with, and between, key stakeholders for the NeoKIP trial to be successful. These included the representatives of the local authorities in the MNHGs, and other communal stakeholders, such as those local representatives of national organisations who have an impact on health care at communal level.

### Local authorities need to be involved

According to study participants, getting the representative (s) of the local authority, that is, the chairperson or vice chairperson (s) of the commune, involved was important to the success of the project. Obtaining the approval of this authority was perceived as a necessity in order to establish the MNHGs and to implement the NeoKIP intervention. These representatives were expected to participate in the final decision-making in all critical activities of the groups.

Study participants perceived that the representatives of the local authority had a stronger voice in society and were in a better position to solve problems than the other MNHG members and/or facilitators. Furthermore, the representatives of the local authorities helped to overcome obstacles, such as dealing with reimbursement requirements of other MNHG members, motivating the participation of members in the MNHG, and improving collaboration with communal stakeholders.

The facilitators’ experience was that the representatives of the local authorities reinforced the project’s messages to a varying extent, depending on how actively engaged they were in the implementation of the project. Having recognized the importance of the representatives of the local authorities, the facilitators and the CHC members of the MNHGs found ways to ensure their support to the group by: 1) personal persuasive communication, for example, calling and discussing MNHG problems, and 2) continuously reporting implementation activities using official channels and/or face-to-face discussions.

“*When we want to do something, we need to ask for permission of the chairperson of the commune. Members of the group can implement only after having their agreement.*” FGD 10 with a MNHG.

### Collaboration of other communal stakeholders is needed

Participants addressed the importance of functional collaboration within the group, and between MNHGs and other stakeholders. In particular, the collaboration of CHC staff and local representatives of country-wide organisations such as the Women’s Union, the Youth Union, and government staff was vital. These stakeholders were described as having interests in local initiatives and as having important roles with regards to improving perinatal health care practices. Thus, their agreement to contribute to the activities of the MNHGs was crucial for the NeoKIP trial.

MNHG members perceived that it was difficult to reach all women through outreach activities. Therefore, to communicate, mobilize and provide services to women, especially to the women of ethnic minorities and those in remote villages, CHC staff needed to collaborate with the population collaborators and the Women’s Union members. In their regular community meetings, these stakeholders could help the CHC staff to communicate health information to community members. Particularly, the Women’s Union was perceived as being central to encouraging and mobilizing women to utilize health services. In MNHGs in areas where the collaboration with these stakeholders could not be established, participants and facilitators recognized the need for support by the local authorities to achieve this cooperation.

“*CHC staff cannot fulfil their tasks if they do not have support from other local stakeholders [such as Women’s Union] in the commune.*” FGD 3 with facilitators

### Incentives to, and motivation of, participants

Different types of incentives and motivating factors were acknowledged to positively influence the implementation of the NeoKIP project.

### Users’ utilization of health services motivates health providers

MNHG members perceived that health care users’ request for better quality of health services was a motivating factor for change. Moreover, community members’ trust and appreciation were recognized as motivating factors for health workers to contribute to the improvement of services provided. The health care providers recognized appreciation to be a reward and they would put in much effort to achieve it.

“*We [staff of the CHC] consider our role more important now [since the implementation of the NeoKIP] because women have a higher awareness of pregnancy and delivery. In the past, no one came here to ask us about these things.*” FGD 7 with a MNHG

### Recognition of benefits among MNHG members is needed

Participants perceived that a supportive working environment was needed for the successful implementation of the NeoKIP intervention. A supportive working environment included opportunities to acquire new knowledge, a culture of sharing information between colleagues and stakeholders, and having good relationships between colleagues. Furthermore, recognition of the intervention’s positive outcomes and working in a multi-stakeholder group were other noted benefits. In the NeoKIP trial, these non-financial incentives were perceived as being crucial to motivate MNHG members to change their behaviour.

“*What is most important is that the local people trust us. Whenever they have any problems with their newborns, they ask for our advice or seek health care in our CHC.*” FGD 15 with a MNHG

### Reimbursement is important but can be balanced by a perceived importance

The MNHG members described NeoKIP as their first project that did not provide reimbursement for their participation. Usually, they could expect to be reimbursed for undertaking any additional project-related activities. Thus, they expected to be paid extra, for example, for outreach visits to communes, and organizing and participating in meetings. Activities related to NeoKIP were considered to be beyond their expected routine tasks and participants therefore considered themselves as having a dual responsibility; one being their regular work, and the other being the activities related to the project. In particular, they argued that members who worked part-time, such as the Women’s Union members and the Village Health Workers, who were normally given a minor allowance by the government, should receive reimbursement for an increase of workload caused by their contribution to a trial like NeoKIP.

“*It [NeoKIP] is a project so it should have a budget for implementation and a budget for reimbursement.*” FGD 16 with a MNHG

MNHG members perceived that contributing their time freely to the project, as a way to improve people’s health, outweighed their requests for additional reimbursement. Moreover, enthusiastic and whole-hearted members were considered to be essential in order for the MNHGs to adhere to what was planned. Participants also stressed the importance of feeling responsible for their work. When the health care providers felt accountable, it increased their engagement in the project-related activities.

“*It’s not like we joined in this group to sit around and shift papers and get a monthly payment from the government. Rather, we work from the heart and we work hard. We love the people in our commune, that’s why we do this.*” FGD 4 with facilitators

### Low health care coverage and utilization

Participants stressed barriers to improve the health care coverage, especially to disadvantaged groups such as ethnic minority groups and those living in resource-scarce settings, which prohibited the MNHGs in their work.

### Acknowledging the disadvantaged groups in society is important to reach the whole population

The differences in health care seeking behaviours and health service accessibility relating to the many ethnic minority groups were depicted in the FGDs. Lack of local language skills of the health care providers was an obstacle for providing evidence-based health care to ethnic minority people. Further, the participants perceived that ethnic minority women had a lack of knowledge about pregnancy and childbirth. These women were also perceived to have low autonomy in health care decision-making. Thus, reaching out to the disadvantaged groups, often living in remote and mountainous areas of the province, was a priority for health care providers. Yet, participants considered that providing evidence-based health services to these people was a challenge.

*“There are many people who deliver at home. They [women in mountainous villages] say that in their village, there is no problem because traditional birth attendants could assist their deliveries. So they do not go to the CHC.”* FGD 5 with a MNHG

### Accessibility and affordability among users influence health care utilization

Participants recognized the inequity in health care accessibility and affordability for people in remote areas. Transportation to health facilities from remote and mountainous areas, where a majority of people of the ethnic minorities lived, was challenging due to great distances and bad roads. The poor infrastructure in these difficult-to-reach areas was further worsened by weather conditions, such as heavy rains and storms. In addition, participants talked about the unaffordability of health care services, especially amongst disadvantaged groups, perceived to have low incomes. When being referred to a higher level of care, people of ethnic minority groups often faced difficulties in dealing with the higher service fees and extra costs for transportation.

*“Home delivery may be unavoidable, because they [women in mountainous villages] cannot use the road to the CHC in extreme weather. For example, if there is a heavy rain like today, the water level rises quickly and the road gets flooded. Therefore, no one can reach the CHC.”* FGD 11 with MNHG

### Lack of resources hinders health service provision

Participants described the shortage of staff, the lack of professional skills amongst the available staff, and the lack of medical equipment as main barriers to provide adequate health services and apply evidence-based health care. Furthermore, the lack of sanitary water and functional rooms for primary health care were cited as obstacles to provide qualified health services.

Consequently, resource shortages, especially in remote and mountainous areas, had a substantial influence on the effectiveness of the MNHGs and the project. Moreover, the heavy workload of MNHG members was a hindrance for them to fully realize the intentions of the project, especially in terms of arranging meetings when all members could be present.

*“Since our commune is no longer classified as a poor commune, it has become more difficult as we don’t get the extra budget support provided for poor communes. We should have that money then we can change things and help people.*” FGD 12 with MNHG

## Discussion

While there is a growing understanding of how and when context influences KT, there are still limited insights into context and KT in LMICs. In this study, we identified three contextual aspects to provide for an enhanced understanding of a community-based project using facilitation as an implementation strategy: (1) the importance of collaboration with and support of key stakeholders to get a project running; (2) the nature of incentives to motivate participants’ efforts; and (3) the need to acknowledge difficulties faced by disadvantaged groups to improve the health care coverage and utilization. These three contextual aspects correspond to features described as important to adapt to in tailored implementation, such as: sectoral, disciplinary, geographic, and cultural factors [9, 10].

In the current study, representatives of the local authorities were perceived to have a crucial role in forming and leading the MNHGs. Not only did the project need to have local authorities’ approval to initiate the work in the MNHG, but also to provide support and coordination when MNHGs planned and implemented their activities. While previous studies report about the need to engage and collaborate with local authorities [29–31], we found that involving local authorities was actually a necessity; the MNHGs depended on the active involvement of representatives of the local authorities, who highly influenced group processes and outcomes.

The heads of the CHCs and representatives of stakeholders in the communes partaking in the MNHGs were in positions to initiate useful collaborative relationships within the group and between MNHGs and other stakeholders. A functioning collaboration, primarily between the local authorities, CHC heads, and representatives of other stakeholders such as the Women’s Union and the Youth Union, affected and fostered the activities of the MNHGs. In having a broad network at all administrative levels of Vietnamese society, members of these unions, particularly the representatives of the Women’s Union, are able to connect with local people and reach most women through communication campaigns [32, 33]. Hence, by collaborating with these unions, the CHC staff could reach every woman in their commune, including disadvantaged groups and women living in remote areas. While the importance of this kind of collaboration is supported by other studies conducted in Vietnam, the effect of a community-based project is often constrained by the fact that this link is rather weak or missing [34–36]. Ensuring the establishment of this collaboration by engaging even more representatives of local organisations in implementation teams like our MNHGs could increase the impact of KT projects in settings similar to Quang Ninh.

In NeoKIP, the participants perceived that project participation traditionally implied extra payment. In addition, MNHG members perceived that they made important contributions, for which they are usually rewarded in Vietnam, and, as such, justify extra payment. Yet, in the NeoKIP trial, the MNHG members were not reimbursed for their participation. The research team argued that to participate in the NeoKIP intervention should be viewed as part of MNHG members’ usual work. While reimbursement can potentially increase participants’ willingness to participate in research and improve retention [37], providing reimbursement in full-scale trials requires considerable research budgets and limits the potential to transfer the findings to real-life health care structures and operations [38, 39]. However, the participants brought up their concern of not being offered financial incentives for participating in the project; the activities in the NeoKIP intervention were perceived as ‘extracurricular work’, and therefore, participants expected to be reimbursed. Still, even without reimbursement, the NeoKIP intervention was accepted by the participants; out of 44 MNHGs in the NeoKIP trial, 43 remained active for the 3-year intervention period [22]. Further, in the current study, we found that the participants recognized the value of non-financial incentives; recognition of their work, receiving appreciation for working in the MNHGs, acquiring new knowledge, sharing information, and being able to strengthen their individual relationships with other group members, were elements which offset not having received extra payment. Similar to our findings, non-financial incentives, such as appreciation from colleagues, job stability, continuous education [40, 41], supportive working environment, and supervision [42, 43], have all been found to be as important as, or even more important, than financial incentives in motivating professional practice [41, 44]. While financial and non-financial incentives related to the category ‘motivation of participants’ effected the facilitation process of NeoKIP trial as reported in our previous publication [23], they were also contextual factors affecting KT. Health providers’ motivation to improve quality of care have repeatedly been reported as an important organizational characteristic [45, 46]. Thus, these incentives could be expected to apply to both the ‘context in which there is a proposed change’ and the ‘facilitation process’. To sum up, our findings support the idea that participants’ perspectives regarding the importance of the project and the potential individual or team benefits could motivate their participation in a KT project, beyond extra payment.

The study participants highlighted difficulties in improving coverage of evidence-based health care, especially among the disadvantaged groups, including the ethnic minority groups and people in rural areas, due to language and logistic barriers. Previously in the NeoKIP trial, we found that disadvantaged groups had a higher risk of neonatal mortality – findings that were linked to the fact that the CHC staff providing health care for these groups had less knowledge on evidence-based neonatal care and that these groups had a considerably longer distance to travel to tertiary-level hospitals [47, 48]. Low health care coverage and poor access to resources did not only influence provision of evidence-based practice but also made up the boundaries for the facilitation process. Also, other reports have stressed that disadvantaged groups living in resource-scarce settings are less likely to receive a quality of care equal to that of resourceful settings [49–51]. Despite the fact that the NeoKIP trial was conducted across a mix of urban and rural communes, the intervention communes achieved a significant reduction of neonatal mortality when compared to the control communes [22]. With an increased focus on communes with disadvantaged groups, the trial might have the potential to achieve an even larger reduction of neonatal mortality.

### Methodological considerations

Although the FGDs were conducted to explore the participants’ experience of facilitation, we found them saturated with regards to context, signifying a secondary analysis [52, 53]. As the aim was to understand if and how the context influences facilitation, the FGDs were conducted only among the facilitation teams in the intervention arm. The secondary analysis, which could provide a more thorough understanding [54], indicated an interplay between the context and facilitation. Factors, that at first sight seemed to relate to either context or facilitation, were found to mutually relate and influence each other. This emphasizes that mechanisms of an intervention are not only passively perceived by participants but they also interact with the intervention activities to co-produce outcomes in a specific context [55]. However, supplementary interviews could supposedly allow further investigation of how these aspects interact. In addition, interviews in the control communes might have provided further information on general aspects of the implementation context in a low- and middle-income setting.

As with any qualitative content analysis, prejudices should be avoided and the best interpretation of the essence of participants’ experience sought [56]. Thus, in this study, the analysis was performed separately by researchers well and little acquainted with the NeoKIP trial and the Vietnamese context, respectively, to support trustworthiness in the analysis process and in the findings [28].

## Conclusions

The findings of this study enhance the understanding of how contextual aspects influenced a KT intervention in a resource-scarce setting. It provides some explanation to why evidence-based knowledge may or may not become everyday practice. Based on the findings, we suggest future KT interventions to apply strategies that engage key stakeholders from the commune and non-governmental organisations working at a local level. Further, KT interventions should identify and communicate non-financial incentives to motivate the participants and make disadvantaged groups a priority. In addition, we suggest future studies should incorporate the examination of context to assist in understanding how context influences KT interventions in LMICs. This will not only help in generating knowledge on the effectiveness of different KT strategies in different settings, but will also guide the development of implementation strategies.
